# Comparative Studies on Polyphenolic Composition, Antioxidant and Antimicrobial Activities of *Schisandra chinensis* Leaves and Fruits

**DOI:** 10.3390/molecules190915162

**Published:** 2014-09-22

**Authors:** Andrei Mocan, Gianina Crișan, Laurian Vlase, Ovidiu Crișan, Dan Cristian Vodnar, Oana Raita, Ana-Maria Gheldiu, Anca Toiu, Radu Oprean, Ioan Tilea

**Affiliations:** 1 Department of Pharmaceutical Botany, Iuliu Hațieganu University of Medicine and Pharmacy, 12 I. Creangă Street, Cluj-Napoca 400010, Romania; E-Mails: mocan.andrei@umfcluj.ro (A.M.); gcrisan@umfcluj.ro (G.C.); 2 Department of Pharmaceutical Technology and Biopharmaceutics, Iuliu Hațieganu University of Medicine and Pharmacy, 12 I. Creangă Street, Cluj-Napoca 400010, Romania; E-Mail: Gheldiu.Ana@umfcluj.ro; 3 Department of Organic Chemistry, Iuliu Hațieganu University of Medicine and Pharmacy, 12 I. Creangă Street, Cluj- Napoca 400010, Romania; 4 Department of Food Science and Technology, University of Agricultural Sciences and Veterinary Medicine, 3-5 Manăştur Street, Cluj-Napoca 400372, Romania; E-Mail: dan.vodnar@usamvcluj.ro; 5 Department of Physics of Nanostructured Materials, National Institute for Research and Development of Isotopic and Molecular Technologies 65-103, Donath Street, Cluj-Napoca 400010, Romania; E-Mail: oana.raita@itim-cj.ro; 6 Department of Pharmacognosy, Iuliu Hațieganu University of Medicine and Pharmacy, 12 I. Creangă Street, Cluj-Napoca 400010, Romania; E-Mail: atoiu@umfcluj.ro; 7 Department of Analytical Chemistry and Instrumental Analysis, Iuliu Hațieganu University of Medicine and Pharmacy, 4 L. Pasteur Street, Cluj-Napoca 400010, Romania; E-Mail: roprean@umfcluj.ro; 8 Family Medicine, Department M3 Clinical Sciences Internal Medicine, University of Medicine and Pharmacy, 38 G. Marinescu Street, Târgu Mures 540139, Romania; E-Mail: ioan.tilea@umftgm.ro

**Keywords:** *Schisandra chinensis*, polyphenols, antioxidants, antimicrobial activity, leaves, fruits

## Abstract

The aim of this paper was to evaluate the antioxidant and antimicrobial activities and the polyphenolic content of *Schisandra chinensis* (Turcz.) Baill. leaves and fruits. The leaves are an important source of flavonoids (35.10 ± 1.23 mg RE/g plant material). Qualitative and quantitative analyses of the polyphenolic compounds were achieved using a HPLC-UV-MS method. The main flavonoid from the leaves was isoquercitrin (2486.18 ± 5.72 μg/g plant material), followed by quercitrin (1645.14 ± 2.12 μg/g plant material). Regarding the fruit composition, the dominant compound there was rutin (13.02 ± 0.21 μg/g plant material), but comparing with the leaves, fruits can be considered a poor source of phenolic compounds. The antioxidant activity was evaluated by DPPH, TEAC, hemoglobin ascorbate peroxidase activity inhibition (HAPX), inhibition of lipid peroxidation catalyzed by cytochrome *c* and EPR spectroscopic assays, revealing a better antioxidant activity for the *S. chinensis* leaves extract. In the antimicrobial assay, *S. chinensis* leaves extract showed efficient activities against the targeted bacteria, being more active than the fruits extract. The results suggest the leaves of *S. chinensis* as a valuable source of antioxidant compounds with significant antioxidant activity.

## 1. Introduction

In recent decades, many herbs and natural compounds have been receiving increased public interest as complementary and alternative medicines [1]. The role of natural compounds in developing new antioxidant agents is well documented [2,3,4], driven by the fact that several studies indicate synthetic antioxidants as possible endocrine disrupters or even carcinogenetic agents [5,6].

Phenolic compounds are one of the most critical ingredients related to free radical scavenging activity in medicinal plants and functional foods [2,7]. They exhibit various biological properties, such as strong antioxidant, cardioprotective, antimutagenic, antibacterial, anti-viral and anti-inflammatory activities [8,9,10]. Considering the important role of phenolic compounds in human health and nutrition, it is mandatory to provide new data on their amounts or variety in medicinal plants and natural foods.

The genus *Schisandra* Michx. (*Schisandraceae*) comprises 23 twining woody species that are widely distributed in East Asia, with a center of diversity found in southeastern and south-central China [11]. The Chinese magnolia vine (Wuweizi, *Schisandra chinesis* (Turcz.) Baill) is a new pharmacopoeial species, introduced into the European phytotherapy just a few years ago, due to its traditional uses in Chinese medicine [12,13]. Its fruits have been used for thousands of years as an astringent, sedative, adaptogenic and tonic agent to treat chronic coughs, spontaneous sweating, palpitation and spermatorrhea [4,14]. Recent studies indicate that *S. chinensis* shows various biological effects, including hepatoprotective, antioxidant [2], neuroprotective [15], immunomodulatory, anticancer [16,17,18], anti-inflammatory, cardioprotective [19,20] and sedative-hypnotic properties [21]. The main bioactive components identified in *Schisandrae chinensis fructus* are dibenzocyclooctadiene derivative lignans or “schisandra lignans” [22]. Many of these lignans were found to exhibit various beneficial biological activities related to the demonstrated pharmacological effects or traditional applications of *S. chinensis*. Biologically active lignans are also present in leaves, but in smaller amounts than in fruits or seeds. The fruits of *S. chinensis* contain, besides lignans, many other important constituents such as organic acids (citric, malic, fumaric and tartaric acid), sugars, vitamic C, vitamin E, phenolic acids, tannins, phytosterols and essential oil [3,22]. Less studied than the fruits, the leaves of *S. chinesis* are used in infusions or as spice, being considered alongside the fruits, an important functional food [23]. The fruits of *Schisandra chinensis* have been used in foods, such as yogurt, jam, fruitcake beverages, wine and other products, as a nutritional and functional ingredient [3]. In China they are considered a flavoring agent and food additive for preparing tea, soup, porridge, *etc.* [14]. The dried fruit is one of the most famous herbal medicines, being known for several thousand years in China. In Japan, *Schisandra* fruit is a widely used component of Kampo medicines and, in the United States, it is a dietary supplement [14,17]. A water extract of the fruits decreased the blood glucose level of normal and alloxan-induced diabetic mice and enhanced the body’s capacity to resist nonspecific stimulation, whilst an ethanol extract showed strong effects on the cardiovascular system [13]. The infusion prepared from leaves, shoots and fruits has stimulatory effects of the body. The decoction and tincture obtained from fruits and seeds are used for treating hepatitis, gastritis, tuberculosis, while the juice and the fresh fruits can increase physical and intellectual working abilities [23].

Compared with the intensive studies on the lignans or their biological activities [24], polyphenolic compounds (phenolic acids and flavonoids) from leaves and fruits and their possible biological effects, have received less attention. Only few reports deal with polyphenolic compounds (anthocyanins), their amounts in the fruits and antioxidant activities [3,25] and no papers focused on the polyphenolic composition of *S. chinensis* leaves could be identified. Due to this fact, further comprehensive studies on polyphenolic compounds and their amounts are essential. A rapid and sensitive HPLC method assisted by mass spectrometry detection was assessed for the determination and quantification of polyphenols in different parts of plant. Considering these aspects, the aim of this paper was to analyse the chemical composition of Romanian harvested *S. chinensis* leaves and fruits and to evaluate the antioxidant and antimicrobial activities, for a better usage and characterization of these functional foods.

## 2. Results and Discussion

### 2.1. HPLC Analysis of Polyphenols

**Figure 1:**
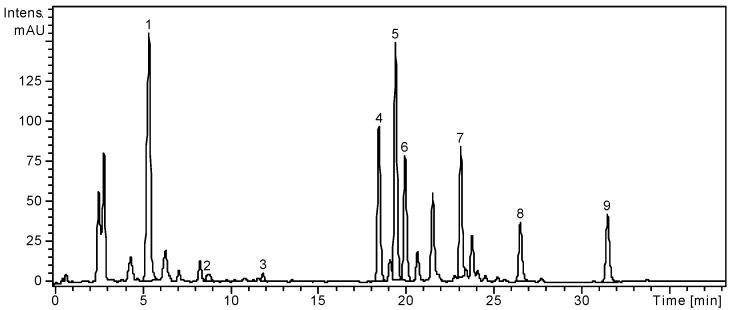
HPLC chromatogram of *S. chinensis* leaves extract.

Notes: Chromatographic conditions were as given in the Experimental section; the identified compounds: 1, Chlorogenic acid; 2, *p*-Coumaric acid; 3, Ferulic acid; 4, Hyperoside; 5, Isoquercitrin; 6, Rutin; 7, Quercitrin; 8, Quercetin; 9, Kaempferol.

**Figure 2:**
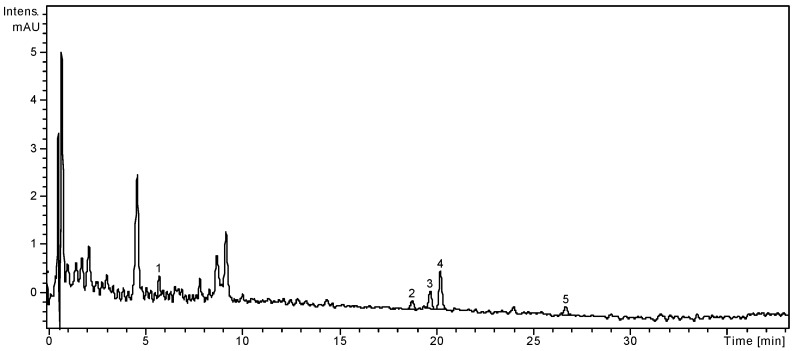
HPLC chromatogram of *S. chinensis* fruits extract.

Notes: Chromatographic conditions were as given in the Experimental section; the identified compounds: 1, Chlorogenic acid; 2, Hyperoside; 3, Isoquercitrin; 4, Rutin; 5, Quercetin.

High performance liquid chromatography (HPLC) assisted by mass spectrometry (MS) detection is an advanced analytical technique that provides high sensitivity and structural information about the analytes. The employed method was developed for the identification and quantification of nineteen phenolic compounds: eight phenolic acids and eleven flavonoids. The analytical method and its applicability were already verified in several other works [1,7,26]. The quantitative determination was performed by the external standard method and the concentrations of the identified phenolic compounds are showed in Table 1 in order of their retention times. HPLC chromatograms of both leaves and fruits of *S. chinensis* are presented in Figure 1 and Figure 2.

**Table 1:** The polyphenolic compounds content in the natural products (μg/g plant material).

Polyphenolic Compounds	*m/z* Value	R_T_ ± SD (min)	*S. chinensis* Leaves	*S. chinensis* Fruits
Gentisic acid	179	3.52 ± 0.04	<0.02	<0.02
Caffeic acid	179	5.60 ± 0.04	<0.02	NF
Chlorogenic acid	353	5.62 ± 0.05	1636.19 ± 4.46	3.26 ± 0.25
*p*-Coumaric acid	163	9.48 ± 0.08	47.74 ± 0.73	<0.02
Ferulic acid	193	12.8 ± 0.10	22.76 ± 0.67	NF
Hyperoside	463	19.32 ± 0.12	1096.61 ± 5.02	1.96 ± 0.02
Isoquercitrin	463	19.60 ± 0.10	2486.18 ± 5.72	6.59 ± 0.07
Rutin	609	20.20 ± 0.15	1365.39 ± 3.32	13.02 ± 0.21
Myricetin	317	21.13 ± 0.12	<0.02	NF
Quercitrin	447	23.64 ± 0.13	1645.14 ± 2.12	NF
Quercetin	301	26.80 ± 0.15	263.25 ± 1.06	1.74 ± 0.04
Kaempferol	285	32.48 ± 0.17	378.27 ± 1.73	NF

Notes: NF—not found, below limit of detection; Values are the mean ± SD (n = 3).

From the analyzed phenolic acids, gentisic, caffeic, chlorogenic, *p*-coumaric and ferulic acids were identified in the ethanolic extract of *S. chinensis* leaves; only chlorogenic, *p*-coumaric and ferulic were quantified with the first being the most abundant (1636.19 ± 4.46 μg/g). Four flavonoid glycosides, hyperoside, isoquercitrin, rutin and quercitrin, and three flavonoid aglycones, myricetin, quercetin and kaempferol were found in the leaves of the Chinese magnolia vine. Among the flavonoid glycosides, isoquercitrin (2486.18 ± 5.72 μg/g) was the main compound (Table 1) and among the free aglycones, the dominant compound was kaempferol (378.27 ± 1.73 μg/g).

The fruits of *S. chinensis* are less rich in polyphenolic compounds. Among the targeted phenolic acids, chlorogenic acid is the only quantified compound (3.26 ± 0.25 μg/g plant material), while gentisic and *p*-coumaric acids were only qualitatively identified. The dominant flavonoid was rutin (13.02 ± 0.21 μg/g), followed by the free aglycone quercetin (1.74 ± 0.04 μg/g). As the literature already specifies, the main phenolic compounds from *Schisandrae chinensis fructus* are not flavonoids or phenolic acids, but rather anthocyanins [3,25]. According to Szopa *et al.* [27], *in vitro* cultures of *S. chinensis* are a potential biotechnological source of therapeutically important phenolic acids. The two cultures obtained from leaf buds (shoot-differentiating and undifferentiated callus culture) were analysed by comparison with the fruits and leaves of the plants growing under natural conditions. Chlorogenic, *p*-coumaric, *p*-hydroxybenzoic, protocatechuic, salicylic and syringic acids were identified in all samples, with higher amounts in fruits and both callus cultures. Chlorogenic acid was the main metabolite in biomass from *in vitro* cultures (226.0 μg/g and 384.3 μg/g, respectively), while in fruits and leaves its content was smaller (19.8 μg/g and 13.7 μg/g, respectively).

Our results showed a higher concentration of chlorogenic acid in leaves, by comparison with the fruits of *S. chinensis* (1636.1 μg/g and 3.2 μg/g, respectively). The differences between the results obtained between the two studies could be due to the different extraction method, time and the solvent used, but also to other factors, both geographical and harvesting related [26].

Polyphenols from two important functional foods, fruits and leaves of *S. chinensis*, were analyzed by using a liquid chromatography coupled with mass spectrometry method. Considering the nineteen target compounds used in this study, some other peaks were not identified. The study revealed significant differences in composition between the fruits and the leaves of *S. chinensis*, indicating that leaves of *S. chinensis* are an important source of flavonoids. A one-way ANOVA test applied on the concentrations values of the identified compounds listed in Table 1 showed that there is a highly significant difference between these two extracts (*p* < 0.001).

### 2.2. Determination of Phenolic Compounds Content

The results of the total flavonoids and total phenolic content of *S. chinensis* extracts are given in Table 2. The total phenolic content (TPC) was expressed as galic acid equivalents (mg GAE/g plant material) and the calculation of total flavonoid content by using a standard curve of rutin and presented as rutin equivalents (mg RE/g plant material).

The leaves of *S. chinensis* contain important amounts of phenolic compounds (62.36 ± 1.38 mg/g plant material) and flavonoids (35.10 ± 1.23 mg/g plant material). The fruits contain significantly lower amounts of polyphenols (9.20 ± 0.43 mg/g plant material) and flavonoids (7.65 ± 0.95 mg/g plant material). Comparing with the results obtained by Chinese authors regarding the fruits concentration in polyphenols and flavonoids, our samples were less rich [28]. A possible explanation could come from the fact that different extraction methods were used. Regarding the total polyphenolic content from leaves, no previous data was found. The presence of biologically active compounds is regulated by a number of factors including plant species, genetic factors, geographical location, tipe of soil, season of harvesting, herb preparation, drying and storage [26].

**Table 2:** The content of total polyphenols and flavonoids in the extracts.

Samples	TPC (mg GAE/g Plant Material)	Flavonoids (mg RE/g Plant Material)
*S. chinensis* leaves	62.36 ± 1.38	35.10 ± 1.23
*S. chinensis* fruits	9.20 ± 0.43	7.65 ± 0.95

Notes: Each value is the mean ± SD of three independent measurements; TPC: Total polyphenols content; GAE: Gallic acid equivalents; RE: rutin equivalents.

### 2.3. Antioxidant Activity Assays

The antioxidant potential of *S. chinensis* extracts was assessed using the DPPH bleaching method, Trolox equivalent antioxidant capacity (TEAC) assay, hemoglobin ascorbate peroxidase activity inhibition (HAPX) assay, the inhibition of lipid peroxidation catalyzed by cytochrome *c* assay and an Electron Paramagnetic Resonance (EPR) spectroscopy method.

The results obtained by the DPPH bleaching assay were presented as quercetin equivalents (Table 3). According to, the fruits extract exhibited a lower antioxidant activity than the leaves. The percentage of DPPH consumption was converted to quercetin equivalents by using a calibration curve (R^2^ = 0.998) with quercetin solutions of 0–12 μM. The higher the rate of DPPH consumption is, the more powerful the antioxidant potential.

**Table 3:** Antioxidant capacity parameters obtained using several methods for studied samples.

Samples	DPPH (µg QE/mg Plant Material)	TEAC (µg TE/mg Plant Material)	HAPX (%)
*S. chinensis* leaves	26.87 ± 0.84	45.97 ± 0.31	31.47 ± 1.36
*S. chinensis* fruits	7.80 ± 0.55	15.95 ± 0.68	28.86 ± 0.21

Notes: Each value is the mean ± SD of three independent measurements; QE: Quercetin equivalents; TE: Trolox equivalents.

The TEAC results are in agreement with the DPPH values and also correlated with the results obtained using other methods. DPPH and TEAC assays are both based on the same principle (free radical scavenging by electron transfer mechanism) and use synthetic radicals which react directly with antioxidants to quantify the antioxidant capacity of the sample.

The enzymatic antioxidant assay (HAPX) measures the capability of the extract active compounds to quench the damage inflicted by hydrogen peroxide upon hemoglobin. Additional information is brought through this assay, since it implies the interaction between antioxidants molecules with a protein, the ferryl hemoglobin species (resulted by the action of hydrogen peroxide on ferric hemoglobin) [29,30].

**Figure 3:**
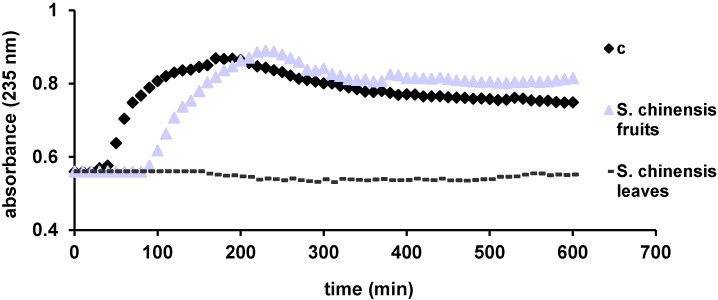
Liposome oxidation by cytochrome *c*, in the presence of the tested samples.

A more physiological method based on peroxidase acticity of cytochrome *c* was used to evaluate the antioxidant activity of the ethanolic extracts. This process monitors the formation of lipid conjugated dienes at 235 nm. According to Yang *et al.*, the inhibition of lipid peroxidation increases with increasing concentrations of flavonoids [31]. Thus, the antioxidant capacity of the tested extracts, reflected in the delay of lipid oxidation, is considered to be based on the same mechanism found in HAPX: the interaction of antioxidants with ferryl, generated in this case in cytochrome *c* [30,32]. Through this assay both extracts demonstrated an antioxidant activity and a good correlation with DPPH and TEAC results (Figure 3). The *S. chinensis* fruits extract delays the peroxidation of lipids about 90 min, while the leaves extract completely blocks the process during the whole experiment (600 min). This is in line with the results of Yang *et al.* [31]. Accordingly, the *S. chinensis* leaves extract exhibits a superior antioxidant activity.

**Figure 4:**
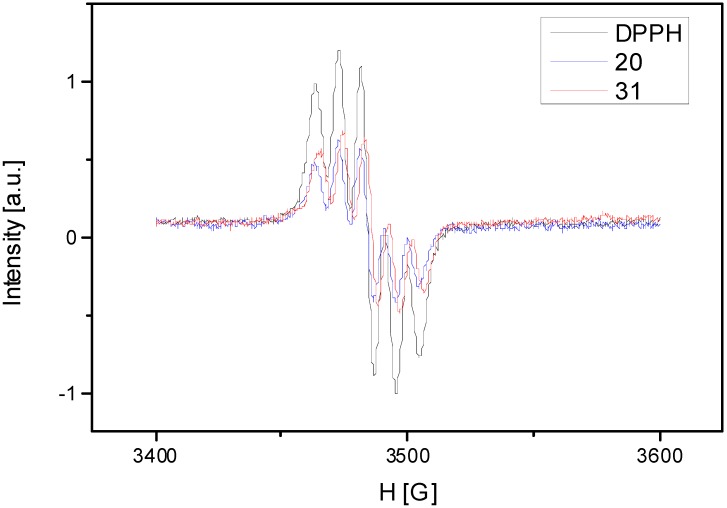
The rate of reaction between antioxidant compounds and DPPH radical.

Notes: 20: *S. chinensis* leaves extract; 31: *S. chinensis* fruits extract.

A common method to evaluate the qualitative antioxidant properties is EPR spectroscopy, using stable free radicals. In this study we made a mixture of free radical (DPPH) and antioxidant extract. The rate of reaction between antioxidant compounds and DPPH radical was monitored by using normalized double integrated residual EPR signal which is correlated with the number of paramagnetic species (Figure 4). One can observe that integral intensity of DPPH in mixture with different antioxidant extracts decreases compared with DPPH solution without antioxidant extract. The EPR spectra presented in the Figure 4 show that we observed a smaller intensity of the signal function of the antioxidant extracts. It represents the oxido-reduction rate of the DPPH radical. Comparing the calculated rates of the both samples, one can observe that *S. chinensis* leaves sample extract has a higher antioxidant capacity than the fruits sample*.* The values of the integral intensity of both samples are represented in Table 4 compared with DPPH.

**Table 4:** The values of integral intensities for the analyzed samples.

DPPH	*S. chinensis* Leaves	*S. chinensis* Fruits
578.85 ± 10.32	167.32 ± 3.24	277.05 ± 7.05

Note: Each value is the mean ± SD of three independent measurements.

The antioxidant potential *S. chinensis* leaves and fruits extracts was investigated using five different tests; the most frequent DPPH and TEAC assays, two complex and physiologically relevant methods based on peroxidase activity of hemoglobin and cytochrome *c* and one EPR spectroscopic method. The antioxidant activity of vegetal extracts is strongly related with their chemical composition. As a peculiarity, *S. chinensis* leaves contain important amounts of flavonoids and chlorogenic acid. High concentrations of flavonoids and chlorogenic acid are reflected in significant scavenging properties [31,33]. In conclusion, the results obtained through antioxidant assays show a good correlation between all methods, with a notable antioxidant activity for the *S. chinensis* leaves. There was a highly significant statistical difference between the analyzed extracts in the DPPH, TEAC and EPR assays (*p* < 0.001), and significant difference in the HAPX method (0.001 < *p* < 0.05).

### 2.4. Antimicrobial Activity Assays

The *in vitro* antibacterial potential of *S. chinensis* extracts against both Gram-positive and Gram-negative bacteria is summarized in Table 5. As can be seen the *S. chinensis* extracts showed important *in vitro* antibacterial activity against all Gram-positive tested bacteria.

**Table 5:** Antibacterial activity of *S. chinensis* extracts and antibiotic against bacterial species tested by disc diffusion assay.

Bacterial Strains	Standard Antibiotic	Inhibition Zone (mm)	
	Gentamicin	*S. chinensis* Fruits	*S. chinensis* Leaves
*Staphylococcus aureus*	2 ± 0.1	4 ± 0.1	9 ± 0.1
*Bacillus subtilis*	6 ± 0.2	7 ± 0.2	11 ± 0.2
*Listeria monocytogenes*	5 ± 0.5	6 ± 0.4	12 ± 0.2
*Escherichia coli*	3 ± 0.3	3 ± 0.1	8 ± 0.2
*Salmonella typhimurium*	6 ± 0.1	6 ± 0.5	9 ± 0.2

Note: Each value is the mean ± SD of three independent measurements.

The strains of *E. coli* and *S. typhimurium* were non-sensitive to the *S. chinensis* fruits extract with a zone of inhibition between 5–6 mm of diameter, comparable with the inhibition zone of gentamicin. On the other hand, *S. chinensis* leaves extract showed efficient antibacterial activities against the targeted bacteria. Thus, the growths of all studied strains were inhibited by *S. chinensis* leaves extract, showing a maximal inhibition zone of 12 mm for *Listeria monocytogenes* strain. The results obtained from the antimicrobial properties can make *S. chinensis* a source for the microbial growth inhibition of Gram positive bacteria. A possible partial explanation is that Gram positive bacteria are more susceptible to the antimicrobial agents than Gram negative bacteria and it is considered to be due to its outer membrane (Cox *et al.* [34]).

The MIC values obtained from antimicrobial tests ranged from 10 µg/mL to >100 µg/mL (Table 6). The results showed that the bacterial strains *S*. *aureus* was more sensitive to *S. chinensis* fruits and leaves extracts with MIC value of 25 µg/mL and 10 µg/mL, respectively; followed by *Bacillus subtilis* with MIC value of 50 µg/mL. Alternativelly, *Listeria monocytogenes, Escherichia coli* and *Salmonella typhimurium* were the less sensitive strains for *S. chinensis* fruits extract, with MIC values of up to 100 µg/mL but sensitive to *S. chinensis* leaves extract with a MIC value of 75 µg/mL. Our results are in agreement with those obtained by Hussain *et al.*, who reported that Gram-positive bacteria are more sensitive to plant essential oils than Gram-negative bacteria [35].

**Table 6:** Minimal Inhibitory Concentration (MIC) of *S. chinensis* extracts.

Bacterial Strains	MIC (µg/mL)	
	*S. chinensis* Fruits	*S. chinensis* Leaves
*Staphylococcus aureus*	25	10
*Bacillus subtilis*	50	50
*Listeria monocytogenes*	>100	75
*Escherichia coli*	>100	75
*Salmonella typhimurium*	>100	75

Note: Each value is the mean ± SD of three independent measurements.

## 3. Experimental Section

### 3.1. Plant Materials and Extraction Procedure

The vegetal material from *S. chinensis* leaves and fruits was purchased from local cultivators from Cluj-Napoca, Romania in the summer of 2013. Voucher specimens (Voucher Nos. 3590 and 3591) were deposited in the Department of Pharmaceutical Botany Herbarium of the Faculty of Pharmacy, “Iuliu Hatieganu” University of Medicine and Pharmacy, Cluj-Napoca, Romania. The vegetal material was air dried at room temperature in shade, separated and grinded to fine powder (300 µm) before the extraction procedures. Twenty grams of each sample were weighed and extracted with 200 mL of 70% ethanol, for 30 min in an ultrasonication bath at 60 °C. The samples were then cooled down and centrifuged at 4500 rpm for 15 min, and the supernatant was recovered [1,7].

### 3.2. Chemical and Instrumentation

Chlorogenic acid, *p*-coumaric acid, caffeic acid, rutin, apigenin, quercetin, isoquercitrin, quercitrin, hyperoside, kaempferol, myricetol, fisetin from Sigma (St. Louis, MO, USA), ferulic acid, sinapic acid, gentisic acid, gallic acid, patuletin, luteolin from Roth (Karlsruhe, Germany), cichoric acid, caftaric acid from Dalton (Toronto, ON, Canada). HPLC grade methanol, ethanol, analytical grade orthophosphoric acid, hydrochloric acid and Folin-Ciocalteu reagent were purchased from Merck (Darmstadt, Germany), hydrogen peroxide, ABTS (2,2'-azinobis-3-ethylbenzotiazoline-6-sulphonic acid), sodium molybdate dihydrate, sodium nitrite, sodium hydroxide, sodium carbonate, sodium acetate trihydrate, and anhydrous aluminum chloride were from Sigma-Aldrich (Steinheim, Germany). DPPH (2,2-diphenyl-1-picrylhydrazyl) and Trolox (6-hydroxy-2,5,7,8-tetramethylchroman-2-carboxylic acid) were obtained from Alfa-Aesar (Karlsruhe, Germany), HRP (horseradish peroxidase) was purchased from Sigma-Aldrich. Bovine hemoglobin was purified following the general protocol of Antonini and Brunori [36]. The met forms of hemoglobin were prepared by ferricyanide treatment as previously described [37]. Liposomes were obtained by suspending 5 mg/mL soybean lecithin (Alfa Aesar) in phosphate buffer followed by sonication and horse heart purified cytochrome *c* from Sigma-Aldrich [30]. All spectrophotometric data were acquired using a Jasco V-530 UV-VIS spectrophotometer (Jasco International Co., Ltd., Tokyo, Japan).

### 3.3. HPLC/MS Analysis

#### 3.3.1. Apparatus and Chromatographic Conditions for the Analysis of Polyphenols

The identification and quantification of polyphenolic compounds was carried out using an Agilent Technologies 1100 HPLC Series system (Agilent, Santa Clara, CA, USA) equipped with G1322A degasser, G13311A binary gradient pump, column thermostat, G1313A autosampler and G1316A UV detector. The HPLC system was coupled with an Agilent 1100 mass spectrometer (LC/MSD Ion Trap SL). For the separation, a reverse-phase analytical column was employed (Zorbax SB-C18 100 × 3.0 mm i.d., 3.5 μm particle); the work temperature was 48 °C. The detection of the compounds was performed on both UV and MS mode. The UV detector was set at 330 nm until 17.5 min, then at 370 nm. The MS system operated using an electrospray ion source in negative mode. The chromatographic data were processed using ChemStation and DataAnalysis software from Agilent. The mobile phase was a binary gradient: methanol and acetic acid 0.1% (v/v). The elution started with a linear gradient, beginning with 5% methanol and ending at 42% methanol, for 35 min; then 42% methanol for the next 3 min [1,7,26]. The flow rate was 1 mL∙min^−1^ and the injection volume was 5 µL.

The MS signal was used only for qualitative analysis based on specific mass spectra of each polyphenol. The MS spectra obtained from a standard solution of polyphenols were integrated in a mass spectra library. Later, the MS traces/spectra of the analyzed samples were compared to spectra from library, which allows positive identification of compounds, based on spectral match. The UV trace was used for quantification of identified compounds from MS detection. Using the chromatographic conditions described above, the polyphenols eluted in less than 40 min (Table 6). Four polyphenols cannot be quantified in current chromatographic conditions due overlapping (caftaric acid with gentisic acid and caffeic acid with chlorogenic acid). However, all four compounds can be selectively identified in MS detection (qualitative analysis) based on differences between their pseudo-molecular mass and MS spectra. For all compounds, the limit of quantification was 0.5 μg/mL, and the limit of detection was 0.1 μg/mL. The detection limits were calculated as minimal concentration producing a reproductive peak with a signal-to-noise ratio greater than three. Quantitative determinations were performed using an external standard method. Calibration curves in the 0.5–50 μg/mL range with good linearity (R^2^ > 0.999) for a five point plot were used to determine the concentration of polyphenols in plant samples [1,7,26].

#### 3.3.2. Identification and Quantification of Polyphenols

The detection and quantification of polyphenols was performed in UV assisted by mass spectrometry detection. Due to peak overlapping, four polyphenol-carboxylic acids (caftaric, gentisic, caffeic, chlorogenic) were determined only based on MS spectra, whereas for the rest of the compounds the linearity of the calibration curves was very good (R^2^ > 0.998), with detection limits in the range of 18 ng/mL to 92 ng/mL. The detection limits were calculated as the minimal concentration yielding a reproductible peak with a signal-to-noise ratio greater than three. Quantitative determinations were performed using an external standard method; retention times were determined with a standard deviation ranging from 0.04 min to 0.19 min (Table 7). For all compounds, the accuracy was between 94.13% and 105.3%. Accuracy was checked by spiking samples with a solution containing each polyphenol in a 10 μg/mL concentration. In all analyzed samples the compounds were identified by comparison of their retention times and recorded electrospray mass spectra with those of standards in the same chromatographic conditions.

**Table 7:** Retention times (R_T_) of polyphenolic compounds (min).

Peak No.	Phenolic Compound	*m/*z	R_T _± SD	Peak No.	Phenolic Compound	*m/z*	RT ± SD
1.	Caftaric acid	311	3.54 ± 0.05	11.	Rutin	609	20.76 ± 0.15
2.	Gentisic acid	153	3.69 ± 0.04	12.	Myricetin	317	21.13 ± 0.12
3.	Caffeic acid	179	6.52 ± 0.04	13.	Fisetin	285	22.91 ± 0.15
4.	Chlorogenic acid	353	6.43 ± 0.05	14.	Quercitrin	447	23.64 ± 0.13
5.	*p*-Coumaric acid	163	9.48 ± 0.08	15.	Quercetin	301	27.55 ± 0.15
6.	Ferulic acid	193	12.8 ± 0.10	16.	Patuletin	331	29.41 ± 0.12
7.	Sinapic acid	223	15.00 ± 0.10	17.	Luteolin	285	29.64 ± 0.19
8.	Cichoric acid	473	15.96 ± 0.13	18.	Kaempferol	285	32.48 ± 0.17
9.	Hyperoside	463	19.32 ± 0.12	19.	Apigenin	279	39.45 ± 0.15
10.	Isoquercitrin	463	20.29 ± 0.10				

Note: SD, standard deviation.

### 3.4. Determination of Total Polyphenols and Flavonoids Content

The total phenolic content (TPC) of the extracts was measured using the Folin-Ciocalteu method with some modifications [7,26]. Two mL from each ethanolic extract were diluted 25 times and then mixed with Folin-Ciocalteu reagent (1 mL) and distilled water (10.0 mL) and diluted to 25.0 mL with a 290 g/L solution of sodium carbonate. The samples were incubated in the dark for 30 min. The absorbance was measured at 760 nm, using a JASCO UV-VIS spectrophotometer. Standard curve was prepared by using different concentrations of gallic acid and the absorbances were measured at 760 nm. TPC values were determined using an equation obtained from the calibration curve of gallic acid graph (R^2^ = 0.999). Total polyphenolic content was expressed as mg gallic acid/g dry material plant (mg GAE/g plant material).

The total flavonoids content was calculated and expressed as rutin equivalents after the method described in the Romanian Pharmacopoeia (Xth Edition) [38]. Each extract (5 mL) was mixed with sodium acetate (5.0 mL, 100 g/L), aluminum chloride (3.0 mL, 25 g/L), and made up to 25 mL in a calibrated flask with methanol. Each solution was compared with the same mixture without reagent. The absorbance was measured at 430 nm. The total flavonoids content values were determined using an equation obtained from calibration curve of the rutin graph (R^2^ = 0.999).

### 3.5. In Vitro Antioxidant Activity Assays

#### 3.5.1. DPPH Bleaching Assay

The DPPH assay provides an easy and rapid way to evaluate potential antioxidants. DPPH free radical method is an antioxidant assay based on electron-transfer that produces a violet solution in ethanol. This free radical, stable at room temperature is reduced in the presence of an antioxidant molecule, giving rise to a yellow solution. The free radical scavenging activity of the ethanolic extracts was measured in terms of hydrogen donating or radical scavenging ability using this method. A stocksolution of 100 µM DPPH was prepared. In a glass cuvette, 2 µL from original extracts were added to 998 µL DPPH solution. The absorbance changes were monitored at 517 nm for 30 min, using a UV-VIS spectrophotometer equipped with a multi-cell holder. The percentage of DPPH consumption in each case was converted to quercetin equivalents using a calibration curve (R^2^ = 0.991) with quercetin standard solutions of 0–12 µM [7,26]. The higher the rate of DPPH consumption is, the more powerful the antioxidant capacity.

#### 3.5.2. TEAC Assay (Trolox Equivalent Antioxidant Capacity)

In the Trolox equivalent antioxidant capacity (TEAC) assay, the antioxidant capacity is reflected in the ability of the natural extracts to decrease the color, reacting directly with the ABTS radical. The latter was obtained by oxidation of ABTS (2,2'-azinobis(3-ethylbenzothiazoline-6-sulfonic acid)) with peroxide, catalyzed by HRP (horseradish peroxidase). Original extracts were diluted 5 times, and 3 µL from the diluted extract were added to 997 µL ABTS solution. The amount of ABTS radical consumed by the tested compound was measured at 735 nm, after 30 min of reaction time. The evaluation of the antioxidant capacity was obtained using the total change in absorbance at this wavelength. The percentage of ABTS consumption was transformed in Trolox equivalents (TE) using a calibration curve (R^2^ = 0.986) with Trolox standard solutions of 0–16 µM [7,26].

#### 3.5.3. Hemoglobin/Ascorbate Peroxidase Activity Inhibition (HAPX) Assay

Inhibition of hemoglobin ascorbate peroxidase activity assay (HAPX) was conducted according to the procedure described by Mot *et al.*, [29]. Hemoglobin was purified according to the Antonini and Brunori protocols [36]. The reaction was triggered by the addition of met hemoglobin (6 µM) to a mixture of ascorbate (160 µM), peroxide (700 µM) and extracts (5 µM) from the stock diluted five times, and it was monitored at 405 nm. This method allows us to evaluate the inhibition of ferryl formation by ascorbate in the presence of the tested compounds. An increase in the time of inhibition reflects the antioxidant capacity of the compound, whereas a decrease, a pro-oxidant effect [37].

#### 3.5.4. Inhibition of Lipid Peroxidation Catalyzed by Cytochrome c

Liposomes were obtained by suspending 5 mg/mL soybean lecithin in phosphate buffer (20 mM, pH 7), followed by sonication for 15 min in an ultrasonic bath (using a Power Sonic 410 device, Thermoline Scientific, Wetherill Park, NSW, Australia). The liposome oxidation experiment was performed at room temperature, for 600 min, in the presence of cytochrome *c* (2 µM) and extracts (5 µL from the diluted extract) by monitoring the absorbance at 235 nm (wavelength specific for liposome oxidation). This process monitors the formation of lipid conjugated dienes at the specified wavelength [30].

#### 3.5.5. EPR Measurements

EPR measurements were performed on a Bruker Elexsys E500 spectrometer (Bruker, Billerica, MA, USA) operating in X band (~9.4 GHz) with 100 kHz modulation frequency, at room temperature. The sample was scanned using the following parameters: centre field, 3360 G; sweep width, 60 G; power, 2 mW; receiver gain, 1 × 10^3^; modulation amplitude, 2 G; time of conversion, 15 ms; time constant, 30.72 ms; sweep time 60 s. A solution of 4.5 mM DPPH was added in liquid samples of antioxidant extracts and quickly mixed with 10 µL of extract and transferred in EPR quartz capillary. The EPR spectra were recorded at different time intervals. The variations of the relative concentration of paramagnetic species were obtained through double integration of experimental spectra using XEPR Bruker software [39].

### 3.6. Determination of Antimicrobial Activity

#### 3.6.1. Microorganisms and Culture Growth

The microorganisms used for antimicrobial activity evaluation were obtained from the University of Agricultural Sciences and Veterinary Medicine Cluj Napoca, Romania. The Gram-positive bacteria *Staphylococcus aureus* (ATCC-25923), *Bacillus subtilis* (ATCC-12228), *Listeria monocytogenes* (ATCC-19115) and gram-negative bacteria *Escherichia coli* (ATCC-25922) and *Salmonella typhimurium* (ATCC-14028). The stock cultures of microorganisms used in this study were maintained on plate count agar slants at 4 °C. Inoculum was prepared by suspending a loop full of bacterial cultures into 10 mL of nutrient agar broth and was incubated at 37 °C for 24 h. About 60 µL of bacterial suspensions, adjusted to 10^6^–10^7^ CFU/mL were taken and poured into Petri plates containing 10 mL sterilized nutrient agar medium. Bacterial suspensions were spread to get a uniform lawn culture [40].

#### 3.6.2. Antimicrobial Activity Assay

Antimicrobial activities of the *S. chinensis* extracts were evaluated by means of agar-well diffusion assay (Bauer *et al.*, 1966) with some modifications [40]. Fifteen millilitres of the molten agar (45 °C) were poured into sterile Petri dishes (Ø 90 mm). Cell suspensions were prepared and 100 µL was evenly spreader onto the surface of the agar plates of Mueller-Hinton agar (Oxoid, Basingstoke, UK). Once the plates had been aseptically dried, 6 mm wells were punched into the agar with a sterile Pasteur pipette. The different extracts (10 mg/mL) were dissolved in dimethylsulfoxide/water (1/9) and 80 µL were placed into the wells and the plates were incubated at 37 °C for 24 h. Gentamicin (25 µL/wells at concentration of 4 µg/mL) was used as positive control for bacteria. Antimicrobial activity was evaluated by measuring the diameter of circular inhibition zones around the well. Tests were performed in triplicate and values are the averages of three replicates [41].

#### 3.6.3. Minimum Inhibitory Concentration

Based on the previous screening the minimum inhibitory concentration (MIC) of both *S. chinensis* extracts was analyzed through the agar-well diffusion method. A bacterial suspension (10^5^–10^6^ CFU/mL) of each tested microorganism was spread on the nutrient agar plate. The wells (6 mm diameter) were cut from agar, and 60 µL of *S. chinensis* extracts dissolved in DMSO at different concentrations (10, 20, 25, 50 75 and 100 µg/mL) were delivered into them. The plates were incubated at 37 °C for 24 h under aerobic conditions that followed by the measurement of the diameter of the inhibition zone expressed in millimeter. MIC was taken from the concentration of the lowest dosed well visually showing no growth after 24 h [42].

### 3.7. Statistical Analysis

A statistical approach was designed and the experimental data were evaluated using one-way analysis of variance (ANOVA), with *p* < 0.05 as threshold for statistical significance. The statistical results confirm the hypothesis that the differences between the results are either not significant (*p* > 0.05), significant (0.001 < *p* < 0.05) or highly significant (*p* < 0.001). The average of multiple measurements (triplicates or more) was listed in the tables together with the standard deviations. Statistical analysis was performed using Excel software package.

## 4. Conclusions

The polyphenolic composition, antioxidant and antimicrobial activities of *S. chinensis* leaves and fruits were analyzed, providing important new data concerning the chemical composition and biological activities of this medicinal plant. Phytochemical investigations reveal *S. chinensis* leaves as a valuable source of flavonoids and chlorogenic acid. Meanwhile, the fruit phytochemical analysis reveals low levels of polyphenols, the main compound being rutin. The results of the antioxidant assays showed a good correlation between several methods, as well as with the content of total polyphenols presenting a relevant antioxidant activity of *S. chinensis* leaves extract. The antimicrobial assays revealed that *S. chinensis* leaves extract has efficient antibacterial activities against targeted bacteria. Thus, the growths of all studied strains were inhibited by *S. chinensis* leaves extract, showing a maximal inhibition zone for *Listeria monocytogenes* strain. Summarizing the results of the present research, we can conclude that *S. chinensis* leaves are a valuable source of flavonoids with important antioxidant and antimicrobial activities.

## Abbreviations

HPLC: high performance liquid chromatography
MS: mass spectrometry
R_T_: retention time
SD: standard deviation
TPC: total phenolic content
GAE: galic acid equivalents
RE: rutin equivalents
TEAC: Trolox equivalent antioxidant capacity
HAPX: hemoglobin ascorbate peroxidase activity
EPR: electron paramagnetic resonance
QE: quercetin equivalents
TE: trolox equivalents
MIC: minimal inhibitory concentration
CFU: colony-forming unit
